# Random Feedback Makes Listeners Tone-Deaf

**DOI:** 10.1038/s41598-018-25518-1

**Published:** 2018-05-08

**Authors:** Dominique T. Vuvan, Benjamin Rich Zendel, Isabelle Peretz

**Affiliations:** 1 Department of Psychology, Skidmore College, 815 N Broadway, Saratoga Springs, NY 12866 United States; 2 International Laboratory for Brain, Music, and Sound Research (BRAMS), 1430 boulevard Mont Royal, Montreal, QC H2V 2J2 Canada; 3 Faculty of Medicine, Memorial University of Newfoundland, 300 Prince Philip Drive, St. John’s, NL A1B3V6 Canada

## Abstract

The mental representation of pitch structure (tonal knowledge) is a core component of musical experience and is learned implicitly through exposure to music. One theory of congenital amusia (tone deafness) posits that conscious access to tonal knowledge is disrupted, leading to a severe deficit of music cognition. We tested this idea by providing random performance feedback to neurotypical listeners while they listened to melodies for tonal incongruities and had their electrical brain activity monitored. The introduction of random feedback was associated with a reduction of accuracy and confidence, and a suppression of the late positive brain response usually elicited by conscious detection of a tonal violation. These effects mirror the behavioural and neurophysiological profile of amusia. In contrast, random feedback was associated with an increase in the amplitude of the early right anterior negativity, possibly due to heightened attention to the experimental task. This successful simulation of amusia in a normal brain highlights the key role of feedback in learning, and thereby provides a new avenue for the rehabilitation of learning disorders.

## Introduction

Tonality is the complex hierarchical structure that governs the organization of pitch in Western music. This structure organizes the 12 chromatic tones into keys, forming a schema that places all the possible tones at varying distances from a central stable pitch known as the tonic. This schema is the basis of the cognition of musical pitch, and informs the perception of tones in musical contexts. The processing of tonality (key) is a core component of music experience and is learned implicitly^1,2^. Despite the implicit nature of its mental representation, listeners can perform tasks that require conscious access to tonal representations. For instance, implicit knowledge about tonal pitch regularities helps listeners to detect out-of-key notes within musical excerpts. This implicit knowledge allows non-musicians to rate the belongingness of probe tones within a tonal context in a manner consistent with music theoretical descriptions of tonality, despite a lack of explicit knowledge of these rules^3^. It is precisely this lack of explicit knowledge that makes access to tonal knowledge prone to experimental manipulation. The main goal of this study was to manipulate conscious access to tonal knowledge in order to model congenital amusia (tone deafness) in neurotypical individuals.

Congenital amusia is a neurodevelopmental disorder causing a lifelong deficit in melodic perception and production that cannot be explained by hearing loss, brain damage, intellectual deficiencies, or lack of music exposure^4^. Recent research on congenital amusia refers to this condition as a disorder of conscious access or awareness^5–7^. Amusics fail to consciously detect tonal violations in melodies. Yet, they seem to have access to tonal knowledge when probed non-consciously^8^. For example, amusics exhibit faster and more accurate responses to a target phoneme when that phoneme is sung on a tonally expected chord compared to an unexpected chord, just like neurotypical controls do^9^. The most compelling evidence comes from studies measuring brain responses to tonal violations. When a pitch falls outside the key of the presented melody, an early negativity is observed in both amusic and control participants^6,10^. This brain response, often called the early right anterior negativity (ERAN), is related to the automatic, non-conscious perception of a tonal hierarchical violation^11,12^ (although it can also be modulated in specific circumstances by attention^13–16^). In contrast, when amusics are asked to detect these pitch violations, the late positivity (i.e., P3, P300, or P600) typically observed in controls is not observed in amusics^6^. This late positivity is related to the conscious, voluntary detection of a tonal violation^17^, and the lack thereof is associated with chance performance^6^. This overall pattern has been repeatedly observed in a number of neurophysiological studies^18–22^. Thus, the amusic deficit seems to be rooted in a lack of conscious access to tonal knowledge that is nevertheless represented by the brain.

Neuroimaging studies of amusia have reported impoverished connectivity with the frontal lobe^8,18,23–25^. Interestingly, both experimental^26–29^ and patient^30,31^ studies of consciousness have reported that the frontal lobe plays a vital role in conscious access to information. In the case of amusia, converging evidence from magnetoencephalography^18^, functional magnetic resonance imaging^24^, voxel-based morphometry^32^, cortical thickness^23^, and diffusion tensor imaging^8^ suggests that disordered connectivity between the right superior temporal gyrus and the right inferior frontal gyrus is the source of the amusic disorder. These studies provide a neurophysiological basis for the lack of conscious access to pitch information. Accordingly, the current view of amusia is that impoverished fronto-temporal connectivity reduces the ability of the frontal lobes to modulate pitch processing in an otherwise normally functioning auditory cortex^18,22^.

Here, we attempted to simulate the disconnection between the auditory cortex, which is taken to represent pitch information (and is associated with the generation of early auditory responses), and the inferior frontal gyrus, which is critical for conscious access to hierarchical representations of pitch (and is associated with the generation of late auditory responses)^11,33–35^, by creating a mismatch between external and internal cues. External cues were manipulated by altering performance feedback during the detection of an out-of-key note in a task originally used by Peretz *et al*.^6^ to study electrical brain responses in amusia. After acquiring baseline measurements of brain activity and task performance without feedback, we interfered with participants’ conscious access to tonal knowledge. This interference was achieved by presenting random feedback on each trial, such that a participant’s responses were reported as 50% “correct” and 50% “incorrect”, regardless of the actual response. To ensure the effects of random feedback were due specifically to its random nature and not simply the occurrence of feedback, a control group of participants was tested. This group went through exactly the same procedure, but received accurate feedback in the blocks following baseline.

For the control group in all blocks and the experimental group during baseline, we predicted that accuracy and confidence would be significantly above chance. Additionally, we expected an ERAN and a late positivity in response to pitch violations (out-of-key notes). These neural responses would indicate a match between the early computation of a tonal violation in the temporal lobe and higher-level conscious integration with prior knowledge in the inferior frontal gyrus. For the experimental group, we predicted that random feedback would induce a top-down perturbation in these cortical interactions. Thus, the administration of random feedback over two blocks of trials should lead to a decrease in both confidence and accuracy as compared to baseline, paralleled by a reduction of the late positive brain responses, while leaving the ERAN unaffected. This pattern of results would mirror the behavioural and neurophysiological expression of amusia. More generally, if we can model the amusic phenotype using false feedback, we could establish whether there is a causal link between unreliable feedback, conscious access to tonal knowledge, and the amusic disorder. If this effect is due specifically to random feedback, we should not observe any change in the behavioural performance or brain activity of control participants across the blocks.

## Methods

### Ethical Approval and Informed Consent

All experimental protocols were approved by the Arts and Sciences Research Ethics Committee (CERAS) at l′Université de Montréal, and the methods were carried out in accordance with this committee’s guidelines. All participants provided informed consent prior to participating in the experiment.

### Participants

Participants were recruited via advertisement on the internet and Université de Montréal campus, and compensated $10/hour for their participation. Participants were recruited first for the experimental group (participants who received false feedback after the baseline), followed by the control group (participants who did not receive false feedback). The experimental and control groups comprised 18 and 22 university students who were matched for age, education, and musical training (Table 1). All participants were non-musicians, with less than 5 years of formal training. Twelve experimental participants and 12 control participants reported no formal musical training, and no participant reported having ever taken music theory or harmony classes. All participants were right-handed, had no known neurological problems, and had normal hearing (according to self-report). Participants were excluded if they failed to score more than 57% correct (2 SD below the mean of a large control sample^36^) in the first block of the experiment. This criterion resulted in the exclusion of three participants who were initially recruited for the control group and five participants who were initially recruited for the experimental group.

**Table 1:** Participant demographics.

	Experimental	Control	Group Comparison
N	18	22	
Gender	9 women	15 women	
Age (years)	—	—	*t*(38) = 0.36, *p* = 0.72
Mean	24.61	24.18	
SD	3.48	3.96	
Range	19–30	19–32	
Education (years)	—	—	*t*(38) = 0.88, *p* = 0.39
Mean	17.78	16.82	
SD	3.59	3.30	
Range	13–27	9–23	
Musical training (years)	—	—	*t*(38) = 0.28, *p* = 0.79
Mean	1.00	1.14	
SD	1.61	1.52	
Range	0–5	0–4	

### Materials

**Figure 1:**
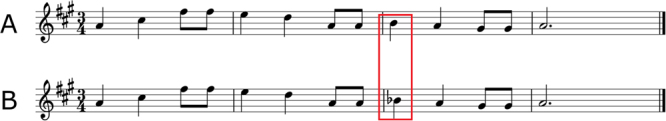
Example of (**A**) an in-key melody and (**B**) the same melody, altered to be out-of-key. The target tone in both melodies is marked in red. Note that performance feedback was presented after the participant’s response, rather than during the melody.

A set of 40 melodies, drawn from a corpus used previously with amusic participants^6,22,37^, served as stimuli. All of these were melodies in a major key and varied in rhythm. Melodies contained between 7 and 15 successive tones ($\bar{{\rm{x}}}$ = 10.3, s = 1.90) and were played at 120 beats per minute (500 ms per beat). The whole set of melodies contained notes ranging in pitch from B4 to C5 (two octaves). Stimulus files are available in MIDI format at https://osf.io/f2tu9/. These melodies were synthesized in four versions, varying with regard to instrumental timbre (guitar or piano) and condition (in-key or out-of-key), resulting in 160 melodies in total. Importantly, the changed pitch always affected the same critical tone, which was 500 ms in duration and fell on the first downbeat in the third bar of the four-bar melody. For in-key melodies, all tones fell within the key of the melody, and for out-of-key melodies, the target tone was shifted by a semitone to fall out of the key of the melody, but remained close in pitch and respected the contour (see Fig. 1 for an example). In an effort to decrease the sensory novelty of out-of-key targets compared to in-key targets, the melodies were presented in eight different keys (A, Bb, B, C, D, Eb, F, or G). Furthermore, ten pitches were used as out-of-key targets (A, Bb, B, C, Db, Eb, E, F, G, Ab), and nine pitches were used as in-key targets (A, Bb, B, C, Db, D, E, F, Gb). Given the significant overlap between conditions, there was no significant difference in frequency of occurrence between in-key and out-of-key targets, *t*(15) = 0.09, *p* = 0.93.

The order of the melodies was set using a pseudorandomization technique to ensure that there was a relatively even distribution of in-key vs. out-of-key melodies and guitar vs. piano timbres across the trials, and that different versions of the same melody were not played back-to-back. The 160 melodies were divided into 20 sets of eight melodies each. Each of these sets contained four in-key melodies and four out-of-key melodies; four of these were produced with a piano sound and the other four with a guitar sound. Only one version (i.e., guitar/piano or in-key/out-of-key) of any of the original 40 melodies could occur in each set.

Participants completed four blocks of 40 trials each. These blocks were created for each participant by randomizing the 8 melodies in each set and then randomly combining the 20 sets into four blocks of five sets. For each melody, participants were asked to judge if the melody contained an anomalous note, and how confident they were of their judgment. Judgments were measured on a four-point scale (1 – anomalous note/sure; 2 – anomalous note/not sure; 3 – no anomalous note/not sure; 4 – no anomalous note/sure). These response choices appeared on the screen during each trial. Participants were required to respond using their right hand.

### Apparatus

Stimuli were presented to participants using a personal computer running Windows XP, with code written and run in MATLAB 2011b, using the Psychophysics toolbox^38,39^. Visual components of the experiment were presented on a Dell Trinitron monitor. The auditory components of the experiment were presented through a pair of Etymotic ER-2 insert earphones connected to a Fireface 800 soundcard, with the volume of the experimental stimuli calibrated to 70 dB SPL. Responses were collected using the computer keyboard.

### Procedure

Prior to the experiment, participants were given written instructions (in addition to the verbal ones just described) and were presented with two examples of each melody type (i.e., melodies containing and not containing an out-of-key note). None of the example melodies were used during the experimental task. Participants were allowed to ask questions and listen to the examples as many times as they wished, until they were comfortable with the task.

### Experimental Group

Once participants were ready to begin, they were informed that they would hear three blocks of melodies (a deception). The first block (Baseline) would constitute practice trials, and they would receive no feedback. Any individual who did not perform significantly above chance (hits − false alarms = 0) was excluded. The second and third blocks (Random 1, Random 2) would constitute test trials, during which they should expect to receive feedback. This feedback would occur in the form of game-show inspired sounds, with “correct” responses being signaled with a ringing bell, and “incorrect” responses being signaled with a low-pitched buzzer. For the experimental group, “correct” vs. “incorrect” feedback was determined randomly (50% trials signaled correct, 50% incorrect), regardless of the actual response of the participant.

After the third block, experimental participants were given a short survey asking whether they had noticed anything strange about the feedback they had received, and if so, what the nature of the alteration might have been. Following this survey, the experimenter debriefed the participant regarding the random feedback they had been receiving. The participant was then informed that we would record a fourth block of trials (Recovery), in which correct feedback would be provided.

### Control Group

The procedure for control participants was identical to the procedure for the experimental group, with a few important changes. First, participants were told that they would hear four blocks of trials (one block of practice – Baseline; three blocks of test trials with correct feedback – Correct 1, Correct 2, Correct 3) from the outset of the experiment. For the second through fourth blocks, “correct” vs. “incorrect” feedback was congruent with participant performance on each trial. Finally, for control participants, there was no break between the third and fourth blocks. Rather, participants were debriefed at the end of the experiment following all four blocks.

The entire experimental session was approximately 1.5 hours in duration.

### Recording and Averaging of Electrical Brain Activity

Encephalography (EEG) was digitized continuously over all four experimental blocks, from 70 active electrodes at a sampling rate of 1024 Hz, using a Biosemi ActiveTwo system. Six electrodes were placed bilaterally at mastoid, inferior ocular, and lateral ocular sites (M1, M2, IO1, IO2, LO1, LO2).

EEG processing was accomplished using Brain Electrical Source Analysis software (BESA, version 5.2). Before the start of the experiment, prototypical eye blinks and horizontal and vertical and eye movements were recorded. A principal component analysis of these prototypical recordings provided a set of components that best explained the eye movements. These components were then decomposed into a linear combination along with topographical components that reflected brain activity. This linear combination allowed the scalp projections of the artifact components to be subtracted from the continuous EEG to minimize ocular contamination such as blinks, vertical and lateral eye movements for each individual average with minimal effects on brain activity^40^. Trials containing excessive noise (>120 µV) at electrodes not adjacent to the eyes (i.e., IO1, IO2, LO1, LO2, FP1, FP2, FPz, FP9, and FP10) were rejected before averaging. On average 87.5% of trials were accepted across all blocks and conditions. To determine whether the number of accepted trials differed across conditions, an ANOVA that included Block (1, 2, 3, 4), and Condition (In-Key, Out-of-Key) was calculated. Neither the main effects of Block and Condition nor their interaction was significant, all *p* > 0.18.

After this correction, continuous EEG was averaged into ERPs based on the onset of the target tone (i.e., in- or out-of-key note). The averaged ERP epoch included 1500 ms of post-target activity. ERP trials were separated for each condition and each block, into eight event-related potentials (ERPs) corresponding to the two conditions (in-key, out-of-key) and four blocks (baseline, random feedback 1, random feedback 2, recovery). Each ERP was corrected using a 50 ms pre-stimulus baseline, band-pass filtered to attenuate frequencies below 0.1 Hz (forward, 6 dB/octave) and above 30 Hz (zero-phase, 12 dB/octave), and referenced to the averaged mastoid. The short 50 ms baseline was used to reduce the amount of noise contained in the baseline period, due to the highly variable but ecologically valid melody stimuli.

## Results

### Data Availability

The datasets generated during and analyzed for the current study are available online at https://osf.io/f2tu9/.

### Statistics

All reported statistical tests were two-tailed. All dependent variable distributions were indistinguishable from a normal distribution, according to the Shapiro-Wilk test.

### Behaviour

#### Accuracy

Accuracy was calculated by subtracting the percentage of false alarms (i.e., incorrect judgment that a melody contains an anomalous note when it does not) from the percentage of hits (i.e., correct judgment that a melody contains an anomalous note) for each block (i.e., hits minus false alarms). As a first step, baseline accuracy was compared between the experimental and control groups. There were no differences in performance between the two groups during the baseline block, *t*(38) = 0.81, *p* = 0.43.

**Figure 2:**
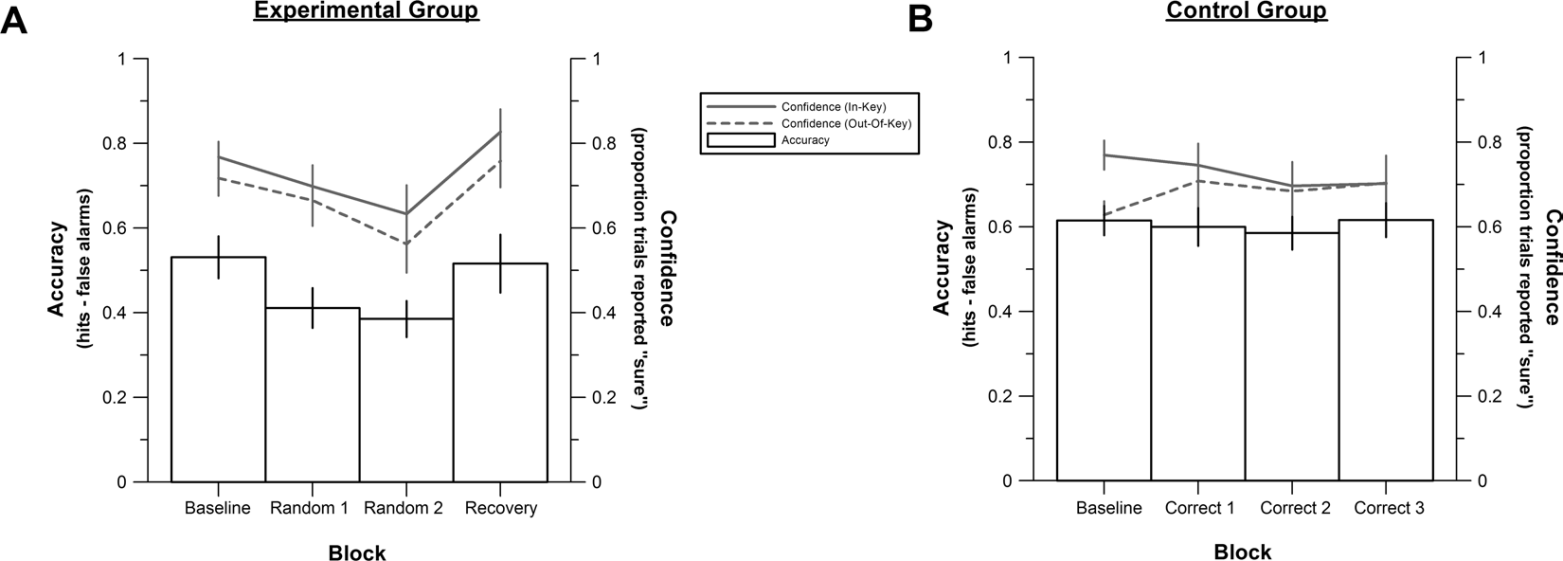
For each experimental block: (**A**) accuracy and confidence in the experimental group; (**B**) accuracy and confidence in the control group.

Accuracy was submitted to mixed effects ANOVA, with Block (1, 2, 3, 4) as a within-subjects factor and Group (Experimental, Control) as a between-subjects factor. Accuracy changed significantly across the four blocks, *F*(3,114) = 7.50, *p* < 0.001, *η*_*p*_^2^ = 0.17, and the control group outperformed the experimental group overall, *F*(1,38) = 5.97, *p* = 0.02, *η*_*p*_^2^ = 0.14. There was also a significant interaction between Block and Group, *F*(3,114) = 3.51, *p* = 0.02, *η*_*p*_^2^ = 0.09, indicating that accuracy changed across blocks for the experimental, but not the control group. Indeed, a quadratic polynomial trend significantly predicted the pattern of accuracy across the four blocks for the experimental group, *F*(1,17) = 14.76, *p* = 0.001, *η*_*p*_^2^ = 0.47, indicating that accuracy fell from baseline during the two random feedback blocks and increased towards baseline levels in the recovery block (Fig. 2). There was no such quadratic effect present in the control group, *F*(1,17) = 1.62, *p* = 0.22, *η*_*p*_^2^ = 0.07.

#### Confidence

Confidence was quantified by calculating the percentage of trials reported as “sure” (regardless of the perceptual judgment) for each block and condition. Confidence was submitted to mixed effects ANOVA, with Block (1, 2, 3, 4) and Condition (In-Key, Out-Of-Key) as within-subjects factors and Group (Experimental, Control) as a between-subjects factor. Participants were more confident in their judgments of melodies in which all notes were in-key compared to melodies containing out-of-key notes, *F*(1,38) = 6.86, *p* = 0.01, *η*_*p*_^2^ = 0.15. Confidence changed across blocks, *F*(3,114) = 4.49, *p* = 0.005, *η*_*p*_^2^ = 0.11, but there was no overall confidence difference between groups, *F*(1,38) < 0.001, *p* = 0.99, *η*_*p*_^2^ < 0.001. However, there was a significant interaction between Block and Group, *F*(3,114) = 3.99, *p* = 0.01, *η*_*p*_^2^ = 0.10, indicating that confidence changed across blocks for the experimental, but not the control group. Indeed, a quadratic polynomial trend significantly predicted the pattern of confidence across the four blocks for the experimental group, *F*(1,17) = 7.45, *p* = 0.01, *η*_*p*_^2^ = 0.31, indicating that confidence fell from baseline during the two random feedback blocks and increased towards baseline levels in the recovery block (Fig. 2). There was no such quadratic effect present in the control group, *F*(1,17) = 0.14, *p* = 0.71, *η*_*p*_^2^ = 0.01. Finally, there was a significant three-way interaction between Group, Block, and Condition, *F*(3,114) = 3.66, *p* = 0.02, *η*_*p*_^2^ = 0.09 (Fig. 2). No other interaction effect was significant, all *F* values < 2.34, all *p* values > 0.07.

Overall, this pattern of results suggests that the random feedback manipulation was effective. Both accuracy and confidence decreased significantly as a result of the introduction of random feedback, and rebounded back towards baseline with the introduction of accurate feedback during the recovery block.

Next, we assessed the effect of false feedback on behaviour in the experimental group. Participants received false feedback when they were told their correct response was incorrect or when they were told their incorrect response was correct. During the random feedback blocks, participants had a 50% chance of being told their response was “correct” or “incorrect” on each trial, regardless of their response. Because of individual differences in task performance, this fixed probability of “correct” vs. “incorrect” feedback led to different participants receiving varying proportions of false feedback. On average, participants in the experimental group received false feedback on 35.17 ± 11.25% of random feedback trials (range = [16%, 56.3%]). In order to assess whether this inter-participant variability in false feedback rate was related to accuracy and confidence, the false feedback rate for each participant was correlated with accuracy and confidence during both random feedback and recovery blocks. There were no significant correlations between the amount of false feedback a participant received and their behavioural performance.

### Event-related Potentials

#### ERAN

**Figure 3:**
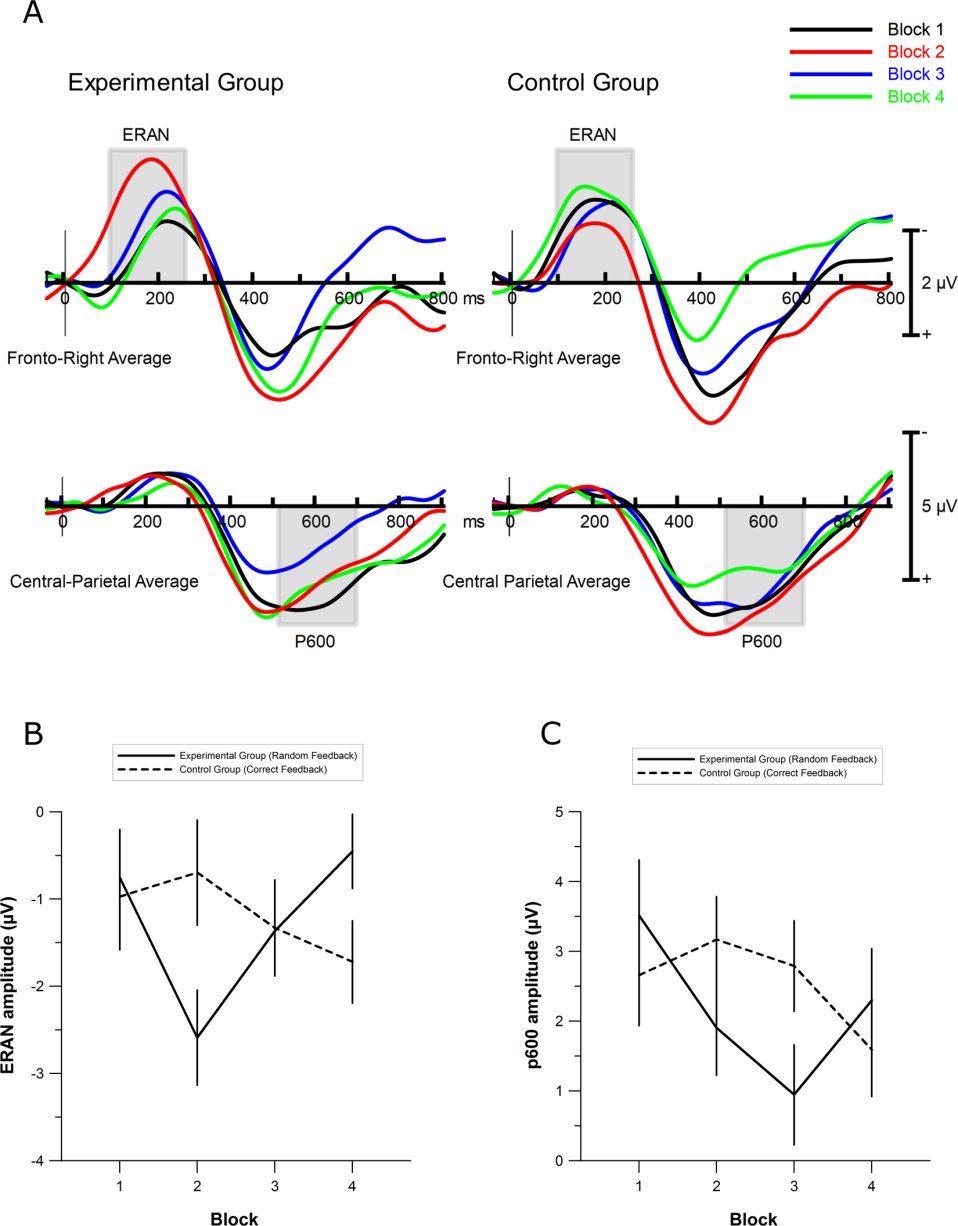
Experimental and control group ERP difference waveforms (out-of-key minus in-key) for the ERAN at electrode F4 (top) and the P3 at electrode POz (bottom). Grey lines highlight the ERAN, P300, and P600. For the experimental group, Block 1 = Baseline, Block 2 = Random Feedback 1, Block 3 = Random Feedback 2, Block 4 = Recovery. For the control group, Block 1 = Baseline, Block 2 = Correct Feedback 1, Block 3 = Correct Feedback 2, Block 4 = Correct Feedback 3.

The ERAN was measured as the mean amplitude difference between out-of-key and in-key notes during the 100–250 ms epoch post-stimulus onset, over twelve right-frontal electrodes (Fz, F2, F4, F6, FCz, FC2, FC4, FC6, Cz, C2, C4 & C6). ERAN amplitude was submitted to mixed effects ANOVA, with Block (1, 2, 3, 4) and Condition (In-Key, Out-Of-Key) as within-subject factors and Group (Experimental, Control) as a between-subjects factor. As can be seen in the difference wave plotted in Fig. 3A, there was greater negativity for the out-of-key note compared to the in-key note, *F*(1,38) = 37.54, *p* < 0.001, *η*_*p*_^2^ = 0.50. The only other significant effect was a three-way interaction between Condition, Block and Group, *F*(3,114) = 2.94, *p* = 0.036, *η*_*p*_^2^ = 0.07.

**Figure 4:**
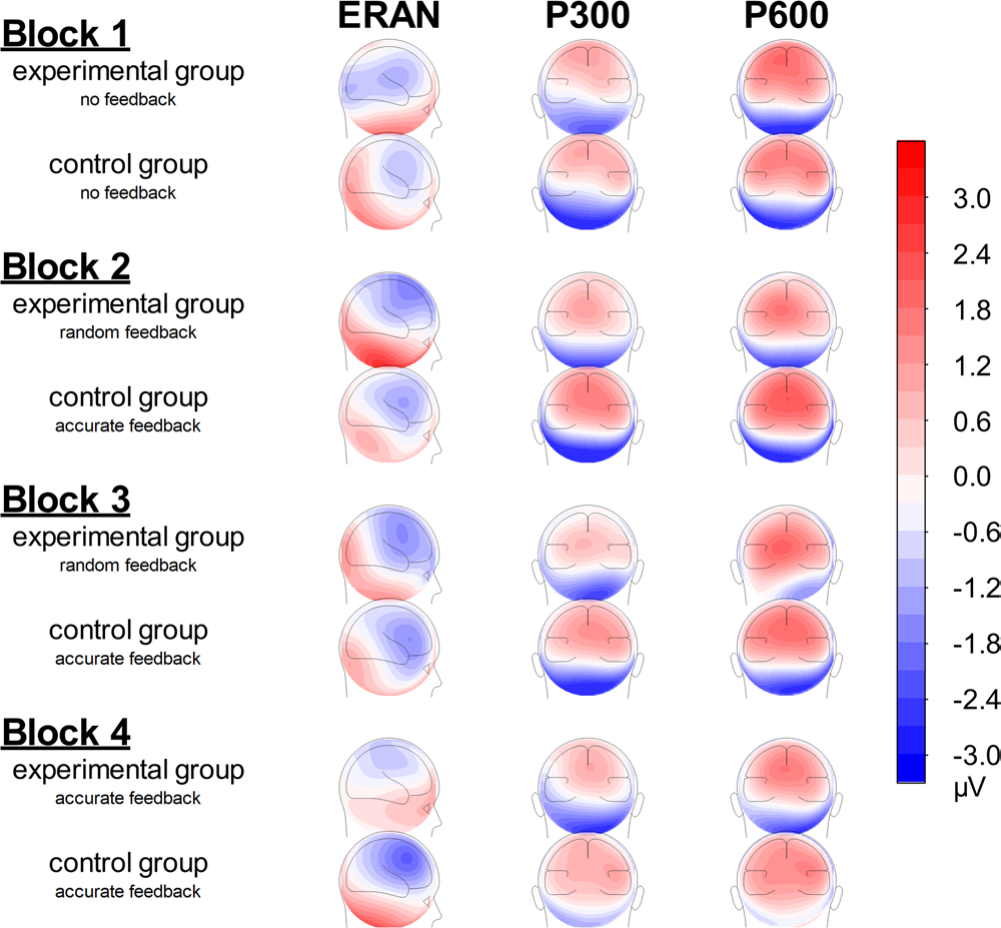
Scalp topographies for the ERAN (100–250 ms), P300 (350–525 ms), and P600 (525–700 ms) in the four experimental blocks.

Follow-up simple two-way interactions revealed a significant Condition by Block interaction for the Random Feedback group, *F*(3,33) = 2.91, *p* = 0.043, *η*_*p*_^2^ = 0.15, but not for the Control group (*p* = 0.61; Fig. 3B). Polynomial decompositions calculated on the difference waves (i.e., out-of-key minus in-key) as a function of Block in the Random Feedback group revealed a quadratic trend, *F*(1,17) = 3.79, *p* = 0.07, *η*_*p*_^2^ = 0.18, with the ERAN being largest during the random feedback blocks (−2.6 µV and −1.4 µV, respectively) compared to blocks 1 and 4 (baseline and recovery blocks; −0.8 µV and −0.5 µV, respectively). The topographical distribution of the difference wave exhibits a right-frontal topography (Fig. 4), suggesting that this component has been correctly identified as an ERAN^11^.

In order to determine if the false feedback rate impacted ERAN amplitude (as was done with accuracy and confidence), the false feedback rate for each participant was correlated with the ERAN amplitude in the random feedback and recovery blocks. ERAN amplitude was not related to the false feedback rate, all *p* > 0.34, although the ERAN increased in amplitude in response to the application of random feedback. Therefore, despite the global effect of random feedback on ERAN amplitude, the ERAN response was insensitive to inter-subject variation in false feedback.

To investigate whether ERAN amplitude was related to task performance a series of bivariate correlations were calculated between accuracy, confidence, and ERAN amplitude, separately for each group. In the experimental group, ERAN amplitude was related to accuracy during the second random feedback block, *r*(18) = −0.49, *p* = 0.04, with increased accuracy being associated with a larger ERAN. No other correlations between ERAN amplitude and accuracy were significant, all *p* > 0.13. Confidence in detecting an out-of-key note was also related to ERAN amplitude in the second random feedback block, *r*(18) = 0.47, p = 0.048, with increased confidence being associated with a larger ERAN amplitude. No other correlations between ERAN amplitude and confidence were significant in the experimental group, all *p* > 0.15. In the control group ERAN amplitude was not related to accuracy nor to confidence in any block, all *p* > 0.19.

#### P3

The late positivity was measured as the mean amplitude difference between out-of-key and in-key notes during the 350–700 ms epoch post-stimulus onset, over six parieto-occipital electrodes (CP1, CPz, CP2, P1, Pz, P2). The electrode montages for the ERAN and positivity were chosen based on previous work^11,17^, and a visual inspection of the overall scalp-topography of the responses. Additionally, we divided the late positivity epoch in half, and separately calculated the mean amplitude for the 350–525 ms epoch and the 525–700 ms epoch. Past research suggests that these two epochs may represent two distinct processing stages. The first epoch might correspond to a traditional P3b, or P300, component representing the orientation of attention to a target sound^17^, whereas the second may correspond to a P600 component, related to the the attentive process of integrating the incongruous note into the musical context^41–44^. Thus we refer to the overall late positivity as the P3, the early epoch of the positivity as the P300, and the late epoch as the P600. Electrode was included as a factor in both analyses, but main effects and interactions with Electrode are not reported because multiple electrode sites were used to obtain a stable estimate of the ERAN and P3 components.

The impact of random feedback on the P300 and P600 was quantified using two separate mixed effects ANOVAs, each with Block and Condition as within-subject factors and Group as a between-subjects factor The topographical distribution of the overall P3 was maximal over midline parietal sites (Fig. 4), suggesting that this component has been correctly identified as a P3^17^.

#### P300

Overall there was a greater early positivity (P300) for the out-of-key notes compared to the in-key note, *F*(1,38) = 36.29, *p* < 0.001, *η*_*p*_^2^ = 0.49. No other effects were significant for this comparison. Next, the effect of false feedback was assessed; the amount of false feedback received by a participant was not related to the amplitude of their P300 component in any block, all *p* > 0.33.

To investigate if P300 amplitude was related to task performance a series of bivariate correlations were calculated between accuracy and P300 amplitude and confidence and P300 amplitude, separately for each group. For the experimental group, P300 amplitude was correlated with accuracy in the baseline block, *r*(18) = 0.62, *p* = 0.006, and the first random feedback block, *r*(18) = 0.48, *p* = 0.045, with increased accuracy associated with a larger P300. This relationship was not present in the second random feedback block nor the recovery block (*p* > 0.36). P300 amplitude was correlated with confidence in detecting an out-of-key note during the baseline block, *r*(18) = 0.47, *p* = 0.049, with a larger P300 being associated with greater confidence. No other correlations between confidence and P300 amplitude were significant for the experimental group (all *p* > 0.28). For the control group, increased accuracy was correlated with increased P300 amplitude in the baseline block, *r*(22) = 0.64, *p* = 0.001, the first feedback block, *r*(22) = 0.45, *p* = 0.034, and the last feedback block, *r*(22) = 0.53, *p* = 0.011. P300 amplitude was associated with increased confidence when identifying an in-key melody during the first feedback block, *r*(22) = 0.46, *p* = 0.03 and the second feedback block, *r*(22) = 0.42, *p* = 0.049. No other relationships between P300 amplitude and confidence were observed for the control group (*p* > 0.07).

#### P600

Overall there was a greater late positivity (P600) for the out-of-key notes compared to the in-key note, *F*(1,38) = 50.82, *p* < 0.001, *η*_*p*_^2^ = 0.57. There was also a marginally significant interaction between Condition, Block, and Group, *F*(3,114) = 2.47, *p* < 0.066, *η*_*p*_^2^ = 0.06. Follow-up simple two-way interactions revealed a marginally significant Block by Condition interaction in the experimental group *F*(3,51) = 2.63, *p* < 0.06, *η*_*p*_^2^ = 0.13, but not in the Control group (*p* = 0.42). Polynomial decompositions calculated on the difference waves (i.e., in-key minus out-of-key) as a function of Block in the experimental group revealed a quadratic trend, *F*(1,17) = 6.20, *p* = 0.02, *η*_*p*_^2^ = 0.27, with the P600 being smallest during the two random feedback blocks (1.91 µV & 0.95 µV, respectively) compared to the baseline and recovery blocks (3.51 µV & 2.30 µV, respectively; see Fig. 3B). Next, the effect of false feedback was assessed; the amount of false feedback received by a participant was not related to the amplitude of their P600 component in any block, all *p* > 0.33.

To investigate if P600 amplitude was related to task performance a series of bivariate correlations were calculated between accuracy and P600 amplitude, and confidence and P600 amplitude, separately for each group. For the experimental group, P600 amplitude was correlated with accuracy in the baseline block, *r*(18) = 0.64, *p* = 0.004, with higher accuracy being associated with a larger P600. In the first random feedback block this relationship weakened, *r*(18) = 0.45, p = 0.06, and then disappeared for the second random feedback and recovery blocks, both *p* > 0.52. P600 amplitude was not correlated with confidence, all *p* > 0.30. For the control group, P600 amplitude was not related to task performance all *p* > 0.12.

Although the P600 occurred during the same timeframe as the N1-P2 for the post-target tone, it was unlikely to be related to that response. For more details see Supplementary Note S1.

### Awareness of Random Feedback (Experimental Group Only)

When asked in the debriefing survey whether they had noticed strange feedback, only seven of the 18 experimental participants answered in the affirmative. To quantify participants’ awareness of the random feedback, a variable was coded such that 0 corresponded to “unaware” (i.e., those who responded that they were unaware or unsure of whether they had noticed strange feedback), and 1 corresponded to “aware”.

#### Behaviour

Awareness of random feedback was not significantly related to any other measured demographic variable (participant age, gender, education, musical training), all *p* > 0.05. To test if awareness of the random feedback impacted accuracy, accuracy data were reanalyzed in the experimental group, using Awareness (aware of random feedback, not aware of random feedback) as a between-subjects factor, and Block as a within-subjects factor. Confidence data were similarly treated, with the addition of Condition (In-Key, Out-Of-Key) as a within-subject variable. There was no effect of Awareness on accuracy, all *F* < 0.59, all *p* > 0.45, nor confidence, all *F* < 1.83, all *p* > 0.19.

#### ERPs

**Figure 5:**
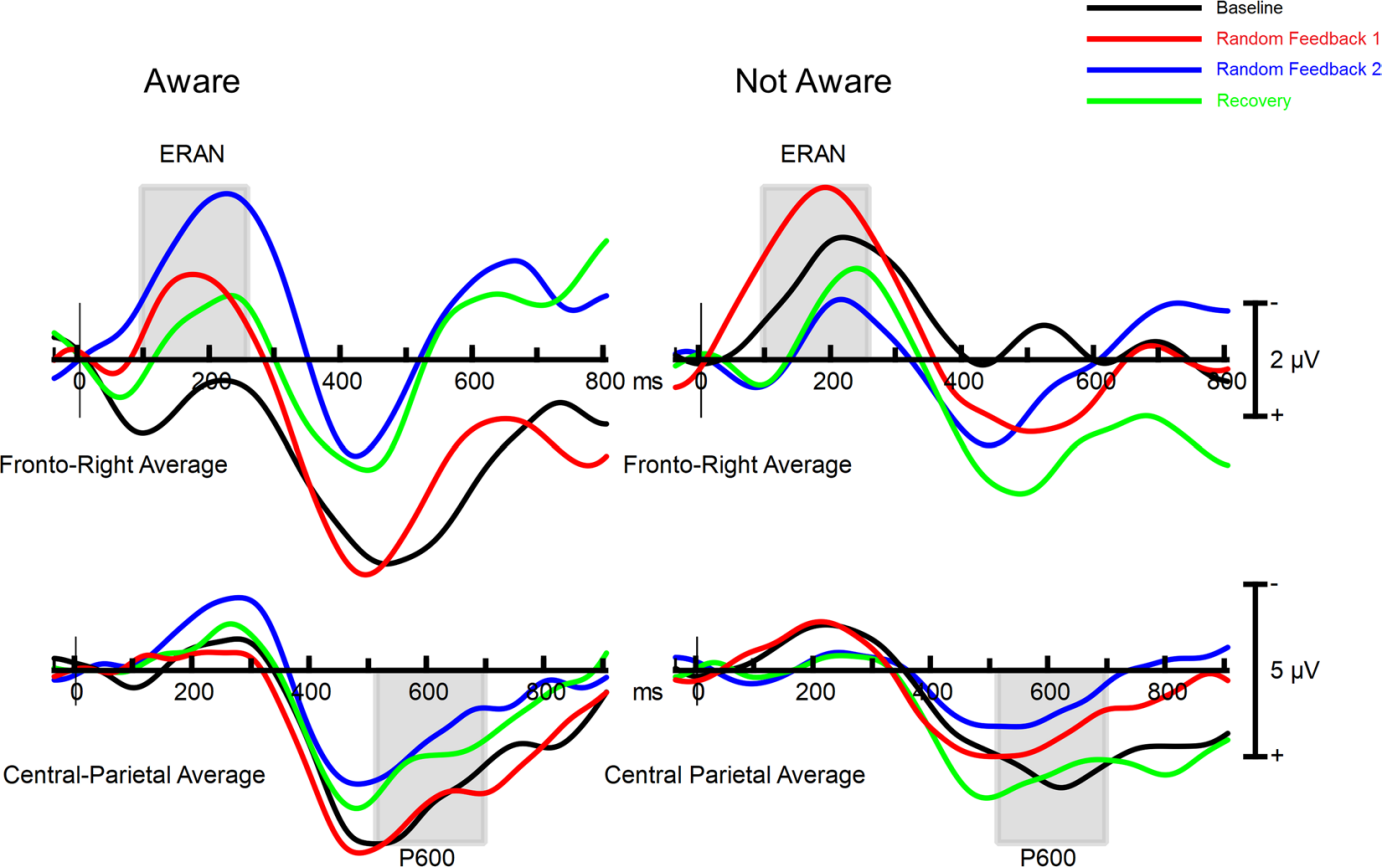
Aware (*n* = 7) and non-aware (*n* = 11) ERP difference waveforms (out-of-key minus in-key) for the ERAN at electrode F4 (top) and the P3 at electrode POz (bottom) in the experimental group. Grey lines highlight the ERAN, P300, and P600.

To test if awareness of the random feedback impacted the ERPs, the amplitude of the ERAN, P300 and P600 were reanalyzed in the experimental group, using Awareness (aware of random feedback, not aware of random feedback) as a between-subjects factor, and block as a within-subjects factor (Fig. 5).

The ERAN was larger in participants who were aware of the random feedback, *F*(1,16) = 8.22, *p* = 0.011, *η*_*p*_^2^ = 0.34. This effect was consistent across all blocks as the Awareness by Block interaction was not significant (*p* = 0.35). In order to determine if the impact of random feedback on the ERAN (see above) was driven by awareness of the feedback, we examined the impact of Block, Condition, and Group. For this analysis, the Group factor compared the control group with experimental group participants who were unaware of random feedback. The interaction between Block and Group was not significant, *F*(3,93) = 2.01, *p* = 0.12, *η*_*p*_^2^ = 0.06. Note that the size of the Group effect for this analysis was similar to the size of the Group effect observed in the omnibus analysis that included all participants (*η*_*p*_^2^ = 0.06 vs. 0.07). The stable effect size, but non-significant interaction suggests that this follow-up analysis was underpowered, due to the nearly half the experimental participants being excluded from this analysis. Given that there was a differential impact of Block on the ERAN on the experimental group as a whole (and the trend was identical when only aware participants were included), it is likely that the impact of random feedback on the ERAN was due to the feedback and not awareness of the feedback.

For the P300, neither the main effect of Awareness nor its interaction with Block was significant (*p* = 0.43 & 0.11, respectively). For the P600, the main effect of Awareness was not significant (*p* = 0.79), nor was its interaction with Block, *F*(1,16) = 2.43, *p* = 0.08, *η*_*p*_^2^ = 0.13.

## Discussion

Random feedback disturbs the musical judgments of ordinary listeners. The presentation of random feedback lowered participants’ accuracy and confidence on a tonal judgment task and decreased the amplitude of the P600 brain response to tonality violations. In contrast, the amplitude of the ERAN increased during random feedback. When correct feedback was finally provided in the final block, behaviour and brain measures recovered toward baseline levels. These changes in behaviour and brain activity were not observed in the control group, who received correct feedback. Thus, the detrimental experimental effects could not be due to the simple occurrence of feedback, and rather can be attributed to the disrupting effects of random feedback specifically.

Previous studies investigating the relation between feedback and performance have predominantly focused on the behavioural effects of (accurate) feedback being present or absent, mostly in the visual modality^45^. The current study extends the limited literature on the effects of random or false feedback on performance from the domain of visual perceptual learning^46,47^ to auditory perception, and confirms previous findings in vision with respect to its disruptive behavioural effects. Furthermore, this study constitutes the first experimental induction of the amusic phenotype in a normal brain. The observed decrease in accuracy and suppression of the P600 mimic the signature of amusia in the current task^22^.

Exposure to random feedback likely increased participants’ attention to the task, which may have activated explicit attempts to access tonal knowledge. Some authors have argued that ERAN amplitude increases with task-directed attention^48^, whereas others have shown that the ERAN is relatively stable, and only changes under very specific manipulations of attention^13–16^. The increase in ERAN amplitude observed in the experimental group might be linked to enhanced attention due to overlap with the N1. N1 amplitude is normally enhanced when attention is directed to the stimulus evoking the N1^49,50^. The random feedback can be seen as introducing noise into participants’ conscious access to tonal knowledge, as demonstrated by declining task performance and suppression of the P600 brain response. The finding that ERAN amplitude continued to increase across both random feedback blocks in participants who were aware of the feedback manipulation supports this idea. These participants likely experienced the most conflict between their tonal knowledge and the feedback, and the ERAN amplitude increase may indicate the strategic drawing of attention to the task in order to amplify conscious access to tonal knowledge.

In contrast, participants who were unaware of the feedback manipulation showed a suppression of this component. This converges with a recent study in which amusics showed an inhibited ERAN in participants with amusia^22^. The decrease in ERAN amplitude for unaware participants may reflect a drawing of attention away from the target tone, due to the random feedback compromising their understanding of the task and therefore their ability to focus on the critical event (the target tone). Importantly, the lack of differences in behaviour between aware and non-aware participants in the experimental group indicates that the noise introduced by random feedback effectively decreased conscious access to tonal knowledge, regardless of the differing strategies used by each group to cope with that noise (as indexed by the differing impact on the ERAN).

Future work will continue to explore this hypothesis by experimentally manipulating awareness of the false feedback manipulation, by titrating the exact amount of false feedback received by participants. These experiments will increase the power to observe graded effects of false feedback over time, which may have gone undetected in the current study due to low inter-subject variability in the percentage of false feedback trials received.

In sum, we successfully simulated the amusic profile using random feedback in typical brains as an experimental model for the conscious access deficit in amusia. The use of this transient experimental model can be compared, for instance, to the use of transcranial magnetic stimulation (TMS) to temporarily “induce” brain lesions^51^. Our successful modeling of the amusic phenotype using false feedback suggests a causal link between unreliable feedback, conscious access to tonal knowledge, and the amusic disorder.

Reliable feedback is a powerful tool for learning. Many studies have shown that learning is more effective when participants attend to (accurate) external feedback^52^. The current results raise the possibility that amusia results from uncertainty due to inappropriate feedback. Since external feedback rarely occurs in ordinary musical activities, the faulty mechanism is more likely to be internal and to arise from the malfunctioning communication between implicitly learned (bottom-up) tonal knowledge and conscious (top-down) access to that intact knowledge^7^. The predictive coding framework provides a useful model for this process.

The predictive coding model sees the brain as a hierarchically organized system in which each level strives to attain an optimal balance between bottom-up sensory information and top-down predictions^53^. Electrophysiological data suggests that this balance is disrupted in amusia, as amusic brains display a normal bottom-up response to tonal deviants (i.e., ERAN), but lack the typical top-down response required to consciously detect those deviants (i.e., P600)^6,22^. In the current non-amusic participants, the enhanced ERAN observed after the administration of random feedback likely represents an amplification of top-down predictive coding. This response could be a neural attempt to amplify the perception of the tonal deviant in order to resolve the anticipated conflict with the potentially false upcoming feedback. In other words, because the external feedback has been inconsistent, the brain attempts to amplify the bottom-up perceptual input to aid in making accurate tonal predictions. Interestingly, the P600, which represents conscious access to the tonal deviant, decreased in amplitude most in the second block of random feedback. This suggests that after abandoning the amplification of the sensory input, the brain recalibrates in order to use the feedback to learn a new (tonal) system. Importantly, this response parallels the behavioural and brain responses observed in amusia.

One clear implication of the current study is that (correct) feedback may be a useful rehabilitative strategy for amusia, as well as other learning disorders, such as dyslexia and prosopagnosia. Indeed, all three learning disorders seem to result from deficient conscious access to intact implicit knowledge. A recent neuroimaging study reveals that adult dyslexics have intact but less accessible phonological representations of speech^54^. Similarly, adults who suffer from prosopagnosia show covert recognition of faces in the absence of overt recognition^55^. Like in amusia, deficient neural communication between sensory and frontal cortices seems to underlie lack of conscious access in dyslexia and prosopagnosia.

However, the case of dyslexia suggests that external feedback, as provided by explicit instruction, is not enough to correct the deficient feedback loop underlying the disorder. Neurofeedback^56^ might be a better strategy. Neurofeedback training is a form of conditioning in which brain activity is rewarded or repressed without requiring conscious access to knowledge. Recent research has successfully applied neurofeedback to children with dyslexia^57^. Future studies of the neural communication between temporal and frontal cortices will help to understand the neurophysiological (and neuroplastic) mechanism responsible for conscious access to knowledge in learning disorders. The manipulation of feedback in a normal brain provides a complementary experimental model to test theories of learning disorders.

## Electronic supplementary material

Supplementary Information
