# Supplementary material for: Random Feedback Makes Listeners Tone-Deaf
Source: Sci Rep. 2018 May 8;8:7283. doi: 10.1038/s41598-018-25518-1 (PMC5940714; doi:10.1038/s41598-018-25518-1)
Supplement: Supplementary file 1 — Supplementary Information [file 41598_2018_25518_MOESM1_ESM.docx]

**Random Feedback Makes Listeners Tone-Deaf**

**Dominique T. Vuvan, Benjamin Rich Zendel and Isabelle Peretz**

**Supplementary Material**

**Supplementary Note S1**

**One concern with the analysis of the P600 is that this waveform overlaps in time with the post-target note. A post-target note always occurred 500 ms after the target tone (in-key or out-of-key note; see Methods – Materials). This tone would evoke an obligatory auditory evoked response (i.e., N1-P2) that could interfere with the P600. This is unlikely, as the P600 analysis focused on the difference waves (out-of-key minus in-key), and the N1-P2 for the post-target note would therefore be subtracted out because the response would be similar for both in-key and out-of-key melodies. To further ensure the P600 was a cognitive response to the out-of-key note and not related to the response evoked by the post-target note we conducted a source analysis to ensure the P600 had separable generators from the N1. This analysis took several steps. First, we considered the in-key target notes during baseline trials from both groups. This baseline block was identical for both groups, and was not impacted by any feedback manipulations. This allowed us to include more participants in the average in order to generate a more stable model to derive source estimates. Next, we fit a symmetrical, 2 dipole model during the N1 epoch (80-120 ms) evoked by the In-key target tones. The model was applied to the in-key tones to avoid interference of the ERAN, which would be elicited by out-of-key tones. The best fit for this model was in the auditory cortex (50 [-50], -29, 14: Talairaich coordinates: transverse temporal gyrus, BA 41). The forward solution from this two dipole model accounted for 82.5% of variance in the scalp-recorded data during the 80-120 ms epoch from in-key trials. This model was then applied to the out-of-key trials during the same epoch (80-120 ms). This forward solution from this model accounted for 88.9% of the variance in scalp-recorded data. The strength of this model suggests the two dipole solution accurately captures the auditory portion of the response. We then examined how much variance the two-dipole model accounted for during the P600 epoch (525-700 ms), for out-of-key notes. The forward solution of this two dipole model accounted for 41.0% of the scalp-recorded data. While holding these two dipoles in place, two more symmetrical dipoles were added to the model, and the best fit for these dipoles was in Brodmann Area 37 (43 [-43],-50, -2: Talairaich coordinates) , just inferior to the temporal parietal junction (TPJ). The forward model of this 4 dipole model accounted for 93.2% of the variance in scalp-recorded data during the P600 epoch. These additional dipoles therefore accounted for an additional 52.3% of the variance in scalp recorded data during the P600 epoch, suggesting that the generators of the P600 were separable from the generators of the N1. Moreover, the source of the P600 dipoles was very close to the temporal-parietal junction. The TPJ has been associated with generation of the P3b responses during tasks that require identifying an auditory target^17^. Overall this source model indicates that the P600 is likely due to the integration of the out-of-key note into the current melodic context by interpreting the out-of-key note in terms of both short-term memory (i.e., veridical expectations; current melodic context) and long-term memory (i.e., schematic expectations; knowledge of tonal structure). Most importantly, the response is not entirely related to the onset of the post-target tone.**
